# Converging Pathways in Autism Spectrum Disorders: Interplay between Synaptic Dysfunction and Immune Responses

**DOI:** 10.3389/fnhum.2013.00738

**Published:** 2013-11-07

**Authors:** Irina Voineagu, Valsamma Eapen

**Affiliations:** ^1^School of Biotechnology and Biomolecular Sciences, University of New South Wales, Sydney, NSW, Australia; ^2^Department of Infant, Child and Adolescent Psychiatry, Academic Unit of Child Psychiatry, University of New South Wales, South West Sydney, Sydney, NSW, Australia

**Keywords:** autism spectrum disorders, immune response, synapses, genomics, gene expression

## Abstract

Autism spectrum disorders (ASD) are highly heritable, yet genetically heterogeneous neurodevelopmental conditions. Recent genome-wide association and gene expression studies have provided evidence supporting the notion that the large number of genetic variants associated with ASD converge toward a core set of dysregulated biological processes. Here we review recent data demonstrating the involvement of synaptic dysfunction and abnormal immune responses in ASD, and discuss the functional interplay between the two phenomena.

## Introduction

**Figure 1:**
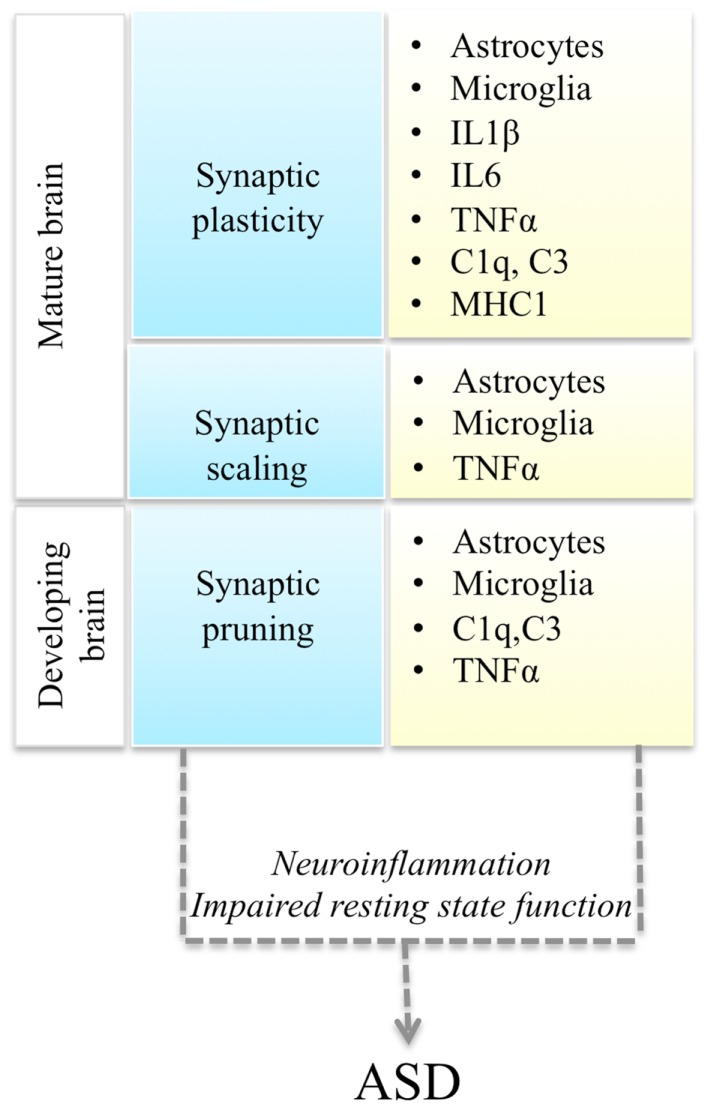
**Interactions between synaptic function and immune molecules and cells, and possible mechanisms leading to ASD**. Multiple aspects of synaptic function (blue boxes) are regulated by astrocytes, microglia, and immune molecules (yellow boxes). Either immune activation during neuroinflammation or impaired resting state activity of immune cells in the brain could impair synaptic function and lead to ASD.

Autism spectrum disorders (ASD) are a spectrum of neurodevelopmental conditions characterized by language deficits, social impairments, and repetitive behaviors (Abrahams and Geschwind, 2008). Typically the disorder is diagnosed around 2–3 years of age and manifests with a regression in acquired language and behavioral skills. However, there are wide variations in the clinical presentation and disease progression. In addition to variable severity of the core symptomatology, ASD patients also present with a variable mix of co-morbid conditions: epilepsy, gastro-intestinal problems, intellectual disability, anxiety, and depression (Kim and Lord, 2013). Mirroring its clinical heterogeneity, ASD is also genetically very heterogeneous (State and Levitt, 2011). Based on the results of genome-wide association (GWAS) studies, candidate gene re-sequencing, and exome-sequencing studies, it is currently estimated that hundreds of genetic variants, including common and rare genetic variants, contribute to the disease (Murdoch and State, 2013). What are the molecular pathways that mediate the phenotypic expression of this myriad of genetic variants into a recognizable triad of symptoms? Here we review recent studies demonstrating a convergence of ASD genetic changes toward two main biological processes: synaptic function and immune responses, and discuss their functional interplay, with a focus on immune modulation of neuronal synapses (Figure 1).

## From Many Genes to Common Biological Processes

Results from genome-wide studies are beginning to confirm the long-held hypothesis that the wide variety of genetic variants associated with ASD ultimately converge on a core set of molecular pathways (Murdoch and State, 2013). It is worth noting that pathway enrichment analyses are inherently limited by our current knowledge of signaling pathways and molecular interactions, and thus the identification of distinct pathways by different studies might partially reflect yet uncharacterized complexity of molecular pathways.

Four recent studies undertook exome-sequencing in several hundreds of parent-child trios in order to identify *de novo* single nucleotide variants (SNVs) and copy number variants (CNVs) associated with the disease (Iossifov et al., 2012; Neale et al., 2012; O’Roak et al., 2012; Sanders et al., 2012). The study by O’Roak et al. found that the most disruptive *de novo* mutations converged onto a highly interconnected beta-catenin/chromatin remodeling protein network, which is involved in neuronal differentiation and synaptic formation (Ille and Sommer, 2005). Iossifov et al. found an enrichment of *de novo* variants in genes encoding proteins associated with the fragile X syndrome protein, FMRP, suggesting an involvement in synaptic plasticity. The study by Neale et al. demonstrated that genes carrying functional *de novo* variants were functionally related to each other and to synaptic genes previously implicated in ASD. Using a network-based analysis of genetic association data, Talkowski et al. (2012) showed that rare *de novo* CNVs occurring in ASD cases affect primarily genes related to synapse development, axon targeting, and neuron motility. Collectively, these studies highlighted the fact that genes containing pathogenic DNA sequence variants in ASD patients affected primarily genes involved in various aspects of synaptic function.

While genetic association studies identify genetic loci potentially implicated in the disease, they do not assess the functional consequences of the associated variants. On the other hand, transcriptome analyses comparing disease and control groups assess gene function (by quantifying mRNA output), but are liable to environmental variations in gene expression, and functional changes unrelated to the disease. Thus ideally, functional genomic studies should simultaneously assess DNA sequence variation and gene expression, in a disease-relevant tissue. However, the scarce availability of post-mortem brain tissue from ASD cases limits the sample size of such studies below appropriate statistical power. Attempting to address this problem, we performed a genome-wide assessment of gene expression in multiple brain regions (frontal cortex, temporal cortex, and cerebellum) from 19 autism cases and 17 unaffected controls, and integrated these results with previous ASD GWAS data (Wang et al., 2009). Using a network-based approach for the analysis of gene expression data we identified two modules of co-expressed genes dysregulated in a large subset of ASD cases (Voineagu et al., 2011). One of these modules was downregulated in ASD brain and was enriched for neuronal genes involved in synaptic function. A second module was upregulated in ASD brain and contained primarily genes functioning in immune and inflammatory responses. To integrate the gene expression results with previously published GWAS data, we performed a pathway enrichment analysis of ASD GWAS data using the two co-expression modules as pre-defined “pathways.” We found that the neuronal genes downregulated in ASD, but not the immune/inflammatory genes, showed an enrichment for genetic association, as measured by a large ASD GWAS study (Wang et al., 2009). These results supported the heritability of synaptic gene dysfunction in ASD and suggested that the upregulation of immune and inflammatory genes is likely environmentally mediated or secondary to the synaptic dysfunction.

Although gene expression analyses of ASD brain are just beginning to emerge, several studies have evaluated gene expression in readily available peripheral tissues (blood and lymphoblast cell lines) from ASD patients (Hu et al., 2006, 2009; Gregg et al., 2008; Enstrom et al., 2009). A common result of these studies was the demonstration of increased expression of immune and inflammatory genes in ASD. Moreover, a comparison of gene expression studies of peripheral tissues in idiopathic autism and related neurodevelopmental disorders showed a convergence of gene expression abnormalities on genes involved in immune responses (Lintas et al., 2012). Interestingly, an analysis of genetic variants nominally associated with ASD found that these variants were enriched in brain expression quantitative trait loci (brain eQTLs), but not lymphoblast eQTLs (Davis et al., 2012). Thus gene expression studies collectively support the concept that (a) immune and inflammatory genes are upregulated in ASD, a phenomenon observed both in the brain and in peripheral tissues, and (b) neuronal synaptic genes are downregulated in ASD brains.

The involvement of synaptic dysfunction and immune responses in ASD had been demonstrated by multiple approaches (Betancur et al., 2009; Pizzarelli and Cherubini, 2011; Wright and Washbourne, 2011; Grabrucker, 2012; Onore et al., 2012; Zoghbi and Bear, 2012; Ebert and Greenberg, 2013), but it was not until large-scale genomic studies that these biological processes could be regarded as points of convergence of the heterogeneous genetic variants underlying ASD.

## Interplay between Brain Immune Processes and Synaptic Function

Microglia, the main resident immune cells in the brain, have been long believed to be active only in response to immune insults, and to exist in a “resting state” in the normal brain. However, this view has dramatically changed over the last decade, and it is becoming increasingly clear that immune cells and molecules play an active role in the normal brain function. Microglia are believed to populate the CNS in the non-vascularized embryonic period and to originate from progenitors from the yolk sac. It has also been proposed that a second wave of microglia, originating from blood monocytes, may populate the CNS during the early postnatal period, a period particularly important for neurodevelopment (Davis and Carson, 2013). Microglia actively survey the brain parenchyma, constantly extending their processes to survey their microenvironment every few hours (Nimmerjahn et al., 2005). Importantly, microglia are required for synaptic pruning during postnatal neurodevelopment (Paolicelli et al., 2011). A recent study (Schafer et al., 2012) demonstrated that microglial synaptic pruning is developmentally regulated and depends on neuronal activity. This process was shown to be mediated by the complement receptor (CR3) pathway, and inhibiting CR3 signaling led to sustained deficits in synaptic connectivity.

Thus immune cells could affect neuronal synaptic function either as a result of their activation during immune responses, or due to a failure of their non-immune roles in the brain (Figure 1). Recent evidence supports the potential involvement of both of these mechanisms in ASD pathogenesis.

Active neuroinflammation has been consistently demonstrated in ASD brains. Prominent activation of microglia (Vargas et al., 2005; Morgan et al., 2010), as well as increased levels of inflammatory cytokines and chemokines [interferon-gamma, IL-1β, IL-6, tumor necrosis factor (TNF)-α] have been documented in post-mortem brain tissue and cerebrospinal fluid from ASD patients (Onore et al., 2012). Recently, activated microglia have also been observed by positron emission tomography in ASD subjects in several brain regions (Suzuki et al., 2013). While it is not clear what is the cause of microglial activation in ASD brain, the cytokines produced by activated microglia have been demonstrated to affect neuronal synaptic function (Onore et al., 2012). TNF-α regulates neuronal cell proliferation and synaptic pruning (Cacci et al., 2005), and modulates synaptic scaling (i.e., the adjustment of synaptic strength for all synapses on a neuronal cell in response to prolonged changes in electrical activity) (Stellwagen and Malenka, 2006). IL-1β regulates long-term potentiation and alters synaptic plasticity (Schneider et al., 1998), while IL-6 has been implicated in behavioral changes associated with maternal immune activation (Patterson, 2009). Mounting evidence suggests that maternal immune activation, particularly during the first and second trimester of pregnancy, may be an important environmental factor in ASD (Onore et al., 2012). Rodent models of maternal immune activation exhibit ASD-like behavioral changes (Patterson, 2009), and the behavioral effects observed in offspring after maternal immune activation appear to be mediated by microglia and IL-6 (Hsiao and Patterson, 2011). In some mouse models increased levels of IL-6 have been sufficient to induce behavioral changes (Onore et al., 2012). Unlike peripheral macrophages, microglia are long-lived, and thus it has been hypothesized that they could maintain an “immunological memory” of an early immune insult, leading to long-term neuronal deficits (Davis and Carson, 2013).

One of the first studies to demonstrate a direct causal relationship between microglial function and a behavioral phenotype, was a mouse model of obsessive-compulsive disorder (Chen et al., 2010). *HOXB8* encodes a homeobox transcription factor expressed in the brain exclusively in bone-marrow-derived microglia. *HOXB8*-null mice exhibit excessive pathological grooming behavior similar to the obsessive-compulsive symptoms of trichotillomania. Chen et al. demonstrated that normal bone marrow transplant could rescue the excessive grooming and hair removal phenotype in the *HOXB8* mutant mouse, and that selective disruption of *HOXB8* in the hematopoietic lineage recapitulates pathological grooming. More recently, a role for non-immune functions of microglia has also been demonstrated in Rett syndrome, a pervasive developmental disorder, belonging to the wider group of ASD. Rett syndrome is caused by loss of function of the methyl-CpG binding protein 2 (*MECP2*) and is characterized by an initial period of normal development of about 5 months followed by deceleration of language development, psychomotor retardation, seizures and loss of social engagement skills (Chahrour and Zoghbi, 2007). It was initially believed that Rett syndrome is primarily due to loss of *MECP2* function in neurons. However several recent studies clearly demonstrated that *MECP2* loss in glial cells impairs neuronal function and contributes to the Rett syndrome symptomatology. *MECP2* deficiency in astrocytes leads to impaired BDNF regulation, cytokine production, and neuronal dendritic arborization (Maezawa et al., 2009). Moreover, *MECP2*-deficient astrocytes are unable to support normal dendritic ramification of wild-type neurons (Ballas et al., 2009). Remarkably, astrocyte-specific expression of *MECP2* in a *MECP2*-null mouse restored the normal neuronal dendritic morphology, improved locomotion, anxiety, and respiratory abnormalities (Lioy et al., 2011). A recent study by Derecki et al. (2012) demonstrated that not only astrocytes but also microglia contribute to the Rett syndrome phenotype. Using irradiation-mediated immune ablation in *MECP2*-null mice, followed by wild-type bone marrow transplantation, this study demonstrated that the wild-type microglia could arrest disease development. In addition, targeted expression of *MECP2* in myeloid cells ameliorated the phenotype in *MECP2*-null mice. These results implicated microglia as important players in the pathophysiology of Rett syndrome, and suggested a potential therapeutic benefit of bone marrow transplantation in Rett syndrome.

## Conclusion and Future Directions

Understanding the core biological processes underlying the clinical and genetic heterogeneity of ASD is as yet in incipient stages. Further advances in elucidating the molecular underpinnings of ASD are expected to result from (a) larger cohort sizes of GWAS and exome-sequencing studies, (b) increased availability of archived post-mortem brain tissue for transcriptome studies, and (c) integrative analyses of genomic, transcriptomic, and epigenomic data.

At the same time, understanding the role of immune cells in regulating synaptic function is also a newly developing field. As discussed above, accumulating evidence supports the notion that immune cells play important roles in normal brain function, outside of neuroinflammation. Of particular relevance to ASD is the role of microglia in synaptic pruning during postnatal brain development, a period that coincides with the onset of ASD symptoms. While it has been demonstrated that increased numbers of activated microglia are present in brain parenchyma of ASD patients (Vargas et al., 2005; Morgan et al., 2010; Suzuki et al., 2013), these studies have not captured the early postnatal development window. Future studies, facilitated by early ASD diagnosis, could shed further light on microglial activation occurs during postnatal brain development and on potential changes in the magnitude of this phenomenon across development and adult life in ASD. Notably, abnormal synaptic density, which could result from a deficit of synaptic pruning, is a feature of several ASD animal models [e.g., increased synaptic density in *Fmr1* KO mice, and decreased synaptic density in Rett syndrome mouse models (Delorme et al., 2013)], but it remains to be demonstrated whether it is also a feature of idiopathic ASD in human brain.

Since microglia and astrocytes have been shown to play a role in synaptic formation and maturation, and mutations in neuronal cell adhesion molecules have been associated with ASD, it is also tempting to speculate that ASD neurons might be particularly vulnerable to immune cell dysfunction in the brain.

Given the large amount of data supporting the role of immune responses in ASD and other neuropsychiatric disorders, advances in deciphering the functional interplay between immune cells and neuronal synaptic function will likely provide vital insights into the mechanisms and potential therapy of neurodevelopmental disorders.

## Conflict of Interest Statement

The authors declare that the research was conducted in the absence of any commercial or financial relationships that could be construed as a potential conflict of interest.

## References

[B1] Abrahams B. S. Geschwind D. H. (2008). Advances in autism genetics: on the threshold of a new neurobiology. Nat. Rev. Genet. 9, 341–355 10.1038/nrg2346 18414403 PMC2756414

[B2] Ballas N. Lioy D. T. Grunseich C. Mandel G. (2009). Non-cell autonomous influence of MeCP2-deficient glia on neuronal dendritic morphology. Nat. Neurosci. 12, 311–317 10.1038/nn.2275 19234456 PMC3134296

[B3] Betancur C. Sakurai T. Buxbaum J. D. (2009). The emerging role of synaptic cell-adhesion pathways in the pathogenesis of autism spectrum disorders. Trends Neurosci. 32, 402–412 10.1016/j.tins.2009.04.003 19541375 PMC10354373

[B4] Cacci E. Claasen J. H. Kokaia Z. (2005). Microglia-derived tumor necrosis factor-alpha exaggerates death of newborn hippocampal progenitor cells in vitro. J. Neurosci. Res. 80, 789–797 10.1002/jnr.20531 15884015

[B5] Chahrour M. Zoghbi H. Y. (2007). The story of Rett syndrome: from clinic to neurobiology. Neuron 56, 422–437 10.1016/j.neuron.2007.10.001 17988628

[B6] Chen S. K. Tvrdik P. Peden E. Cho S. Wu S. Spangrude G. (2010). Hematopoietic origin of pathological grooming in Hoxb8 mutant mice. Cell 141, 775–785 10.1016/j.cell.2010.03.055 20510925 PMC2894573

[B7] Davis D. S. Carson M. J. (2013). “An introduction to CNS-resident microglia: definitions, assays, and functional roles in health and disease,” in Neural-Immune Interactions in Brain Function and Alcohol Related Disorders, eds Cui C. Grandison L. Noronha A. (New York: Springer), 3–29 10.1007/978-1-4614-4729-0_1

[B8] Davis L. K. Gamazon E. R. Kistner-Griffin E. Badner J. A. Liu C. Cook E. H. (2012). Loci nominally associated with autism from genome-wide analysis show enrichment of brain expression quantitative trait loci but not lymphoblastoid cell line expression quantitative trait loci. Mol. Autism 3, 3 10.1186/2040-2392-3-3 22591576 PMC3484025

[B9] Delorme R. Ey E. Toro R. Leboyer M. Gillberg C. Bourgeron T. (2013). Progress toward treatments for synaptic defects in autism. Nat. Med. 19, 685–694 10.1038/nm.3193 23744158

[B10] Derecki N. C. Cronk J. C. Lu Z. Xu E. Abbott S. B. Guyenet P. G. (2012). Wild-type microglia arrest pathology in a mouse model of Rett syndrome. Nature 484, 105–109 10.1038/nature10907 22425995 PMC3321067

[B11] Ebert D. H. Greenberg M. E. (2013). Activity-dependent neuronal signalling and autism spectrum disorder. Nature 493, 327–337 10.1038/nature11860 23325215 PMC3576027

[B12] Enstrom A. M. Lit L. Onore C. E. Gregg J. P. Hansen R. L. Pessah I. N. (2009). Altered gene expression and function of peripheral blood natural killer cells in children with autism. Brain Behav. Immun. 23, 124–133 10.1016/j.bbi.2008.08.001 18762240 PMC2636576

[B13] Grabrucker A. M. (2012). Environmental factors in autism. Front. Psychiatry 3:118 10.3389/fpsyt.2012.00118 23346059 PMC3548163

[B14] Gregg J. P. Lit L. Baron C. A. Hertz-Picciotto I. Walker W. Davis R. A. (2008). Gene expression changes in children with autism. Genomics 91, 22–29 10.1016/j.ygeno.2007.09.003 18006270

[B15] Hsiao E. Y. Patterson P. H. (2011). Activation of the maternal immune system induces endocrine changes in the placenta via IL-6. Brain Behav. Immun. 25, 604–615 10.1016/j.bbi.2010.12.017 21195166 PMC3081363

[B16] Hu V. W. Frank B. C. Heine S. Lee N. H. Quackenbush J. (2006). Gene expression profiling of lymphoblastoid cell lines from monozygotic twins discordant in severity of autism reveals differential regulation of neurologically relevant genes. BMC Genomics 7:118 10.1186/1471-2164-7-118 16709250 PMC1525191

[B17] Hu V. W. Nguyen A. Kim K. S. Steinberg M. E. Sarachana T. Scully M. A. (2009). Gene expression profiling of lymphoblasts from autistic and nonaffected sib pairs: altered pathways in neuronal development and steroid biosynthesis. PLoS ONE 4:e5775 10.1371/journal.pone.0005775 19492049 PMC2685981

[B18] Ille F. Sommer L. (2005). Wnt signaling: multiple functions in neural development. Cell. Mol. Life Sci. 62, 1100–1108 10.1007/s00018-005-4552-2 15928805 PMC11139179

[B19] Iossifov I. Ronemus M. Levy D. Wang Z. Hakker I. Rosenbaum J. (2012). De novo gene disruptions in children on the autistic spectrum. Neuron 74, 285–299 10.1016/j.neuron.2012.04.009 22542183 PMC3619976

[B20] Kim S. H. Lord C. (2013). “The behavioral manifestations of autism spectrum disorders,” in The Neuroscience of Autism Spectrum Disorders, ed. Buxbaum J. D. (New York: Elsevier), 25–37

[B21] Lintas C. Sacco R. Persico A. M. (2012). Genome-wide expression studies in autism spectrum disorder, Rett syndrome, and Down syndrome. Neurobiol. Dis. 45, 57–68 10.1016/j.nbd.2010.11.010 21130877

[B22] Lioy D. T. Garg S. K. Monaghan C. E. Raber J. Foust K. D. Kaspar B. K. (2011). A role for glia in the progression of Rett’s syndrome. Nature 475, 497–500 10.1038/nature10214 21716289 PMC3268776

[B23] Maezawa I. Swanberg S. Harvey D. LaSalle J. M. Jin L. W. (2009). Rett syndrome astrocytes are abnormal and spread MeCP2 deficiency through gap junctions. J. Neurosci. 29, 5051–5061 10.1523/JNEUROSCI.0324-09.2009 19386901 PMC3436907

[B24] Morgan J. T. Chana G. Pardo C. A. Achim C. Semendeferi K. Buckwalter J. (2010). Microglial activation and increased microglial density observed in the dorsolateral prefrontal cortex in autism. Biol. Psychiatry 68, 368–376 10.1016/j.biopsych.2010.05.024 20674603

[B25] Murdoch J. D. State M. W. (2013). Recent developments in the genetics of autism spectrum disorders. Curr. Opin. Genet. Dev. 23, 310–315 10.1016/j.gde.2013.02.003 23537858

[B26] Neale B. M. Kou Y. Liu L. Ma’ayan A. Samocha K. E. Sabo A. (2012). Patterns and rates of exonic de novo mutations in autism spectrum disorders. Nature 485, 242–245 10.1038/nature11011 22495311 PMC3613847

[B27] Nimmerjahn A. Kirchhoff F. Helmchen F. (2005). Resting microglial cells are highly dynamic surveillants of brain parenchyma in vivo. Science 308, 1314–1318 10.1126/science.1110647 15831717

[B28] Onore C. Careaga M. Ashwood P. (2012). The role of immune dysfunction in the pathophysiology of autism. Brain Behav. Immun. 26, 383–392 10.1016/j.bbi.2011.08.007 21906670 PMC3418145

[B29] O’Roak B. J. Vives L. Girirajan S. Karakoc E. Krumm N. Coe B. P. (2012). Sporadic autism exomes reveal a highly interconnected protein network of de novo mutations. Nature 485, 246–250 10.1038/nature10989 22495309 PMC3350576

[B30] Paolicelli R. C. Bolasco G. Pagani F. Maggi L. Scianni M. Panzanelli P. (2011). Synaptic pruning by microglia is necessary for normal brain development. Science 333, 1456–1458 10.1126/science.1202529 21778362

[B31] Patterson P. H. (2009). Immune involvement in schizophrenia and autism: etiology, pathology and animal models. Behav. Brain Res. 204, 313–321 10.1016/j.bbr.2008.12.016 19136031

[B32] Pizzarelli R. Cherubini E. (2011). Alterations of GABAergic signaling in autism spectrum disorders. Neural Plast. 2011, 297153 10.1155/2011/297153 21766041 PMC3134996

[B33] Sanders S. J. Murtha M. T. Gupta A. R. Murdoch J. D. Raubeson M. J. Willsey A. J. (2012). De novo mutations revealed by whole-exome sequencing are strongly associated with autism. Nature 485, 237–241 10.1038/nature10945 22495306 PMC3667984

[B34] Schafer D. P. Lehrman E. K. Kautzman A. G. Koyama R. Mardinly A. R. Yamasaki R. (2012). Microglia sculpt postnatal neural circuits in an activity and complement-dependent manner. Neuron 74, 691–705 10.1016/j.neuron.2012.03.026 22632727 PMC3528177

[B35] Schneider H. Pitossi F. Balschun D. Wagner A. del Rey A. Besedovsky H. O. (1998). A neuromodulatory role of interleukin-1beta in the hippocampus. Proc. Natl. Acad. Sci. U.S.A. 95, 7778–7783 10.1073/pnas.95.13.7778 9636227 PMC22755

[B36] State M. W. Levitt P. (2011). The conundrums of understanding genetic risks for autism spectrum disorders. Nat. Neurosci. 14, 1499–1506 10.1038/nn.2924 22037497 PMC3940335

[B37] Stellwagen D. Malenka R. C. (2006). Synaptic scaling mediated by glial TNF-alpha. Nature 440, 1054–1059 10.1038/nature04671 16547515

[B38] Suzuki K. Sugihara G. Ouchi Y. Nakamura K. Futatsubashi M. Takebayashi K. (2013). Microglial activation in young adults with autism spectrum disorder. JAMA Psychiatry 70, 49–58 10.1001/jamapsychiatry.2013.272 23404112

[B39] Talkowski M. E. Maussion G. Crapper L. Rosenfeld J. A. Blumenthal I. Hanscom C. (2012). Disruption of a large intergenic noncoding RNA in subjects with neurodevelopmental disabilities. Am. J. Hum. Genet. 91, 1128–1134 10.1016/j.ajhg.2012.10.016 23217328 PMC3516594

[B40] Vargas D. L. Nascimbene C. Krishnan C. Zimmerman A. W. Pardo C. A. (2005). Neuroglial activation and neuroinflammation in the brain of patients with autism. Ann. Neurol. 57, 67–81 10.1002/ana.20315 15546155

[B41] Voineagu I. Wang X. Johnston P. Lowe J. K. Tian Y. Horvath S. (2011). Transcriptomic analysis of autistic brain reveals convergent molecular pathology. Nature 474, 380–384 10.1038/nature10110 21614001 PMC3607626

[B42] Wang K. Zhang H. Ma D. Bucan M. Glessner J. T. Abrahams B. S. (2009). Common genetic variants on 5p14.1 associate with autism spectrum disorders. Nature 459, 528–533 10.1038/nature07999 19404256 PMC2943511

[B43] Wright G. J. Washbourne P. (2011). Neurexins, neuroligins and LRRTMs: synaptic adhesion getting fishy. J. Neurochem. 117, 765–778 10.1111/j.1471-4159.2010.07141.x 21155806 PMC3066302

[B44] Zoghbi H. Y. Bear M. F. (2012). Synaptic dysfunction in neurodevelopmental disorders associated with autism and intellectual disabilities. Cold Spring Harb. Perspect. Biol. 4, ii:a009886 10.1101/cshperspect.a009886 22258914 PMC3282414

